# Supplementary material for: Robust changes in global subtropical circulation under greenhouse warming
Source: Nat Commun. 2024 Jan 2;15:96. doi: 10.1038/s41467-023-44244-5 (PMC10762120; doi:10.1038/s41467-023-44244-5)
Supplement: Supplementary file 1 — Supplementary Information [file 41467_2023_44244_MOESM1_ESM.pdf]

Supplementary Information for

Robust changes in global subtropical circulation under greenhouse warming

Shijie Zhou, Ping Huang, Lin Wang, Kaiming Hu, Gang Huang and Peng Hu

Table S1 CMIP6 models and experiments used in this study.

Model name	historical & SSP5-8.5	piControl & abrupt-4xCO2	amip, amip-p4K, amip-4xCO2 & amip-future4K
ACCESS-CM2	X	X	
ACCESS-ESM1-5	X	X	
AWI-CM-1-1-MR		X	
BCC-CSM2-MR	X	X	X
BCC-ESM1		X	
CAMS-CSM1-0	X	X	
CESM2	X	X	X
CESM2-FV2		X	
CESM2-WACCM	X		
CESM2-WACCM-FV2		X	
CIESM		X	
CMCC-CM2-SR5		X	
CNRM-CM6-1	X	X	X
CNRM-CM6-1-HR	X		
CNRM-ESM2-1	X	X	
CanESM5	X	X	X
CanESM5-CanOE	X		
E3SM-1-0		X	
EC-Earth3	X		
EC-Earth3-Veg	X	X	
FGOALS-f3-L	X	X	
FGOALS-g3	X	X	
GFDL-CM4	X	X	X
GFDL-ESM4	X	X	
GISS-E2-1-G	X		
GISS-E2-1-H	X		
HadGEM3-GC31-LL	X		X
INM-CM4-8	X	X	
INM-CM5-0	X	X	
IPSL-CM6A-LR	X	X	X
KACE-1-0-G	X		
MIROC-ES2L	X	X	
MIROC6	X	X	X
MPI-ESM-1-2-HAM		X	
MPI-ESM1-2-HR	X	X	
MPI-ESM1-2-LR	X	X	
MRI-ESM2-0	X	X	X
NESM3	X		
NorESM2-LM	X	X	

NorESM2-MM	X		
SAM0-UNICON		X	
TaiESM1	X		X
UKESM1-0-LL	X	X	

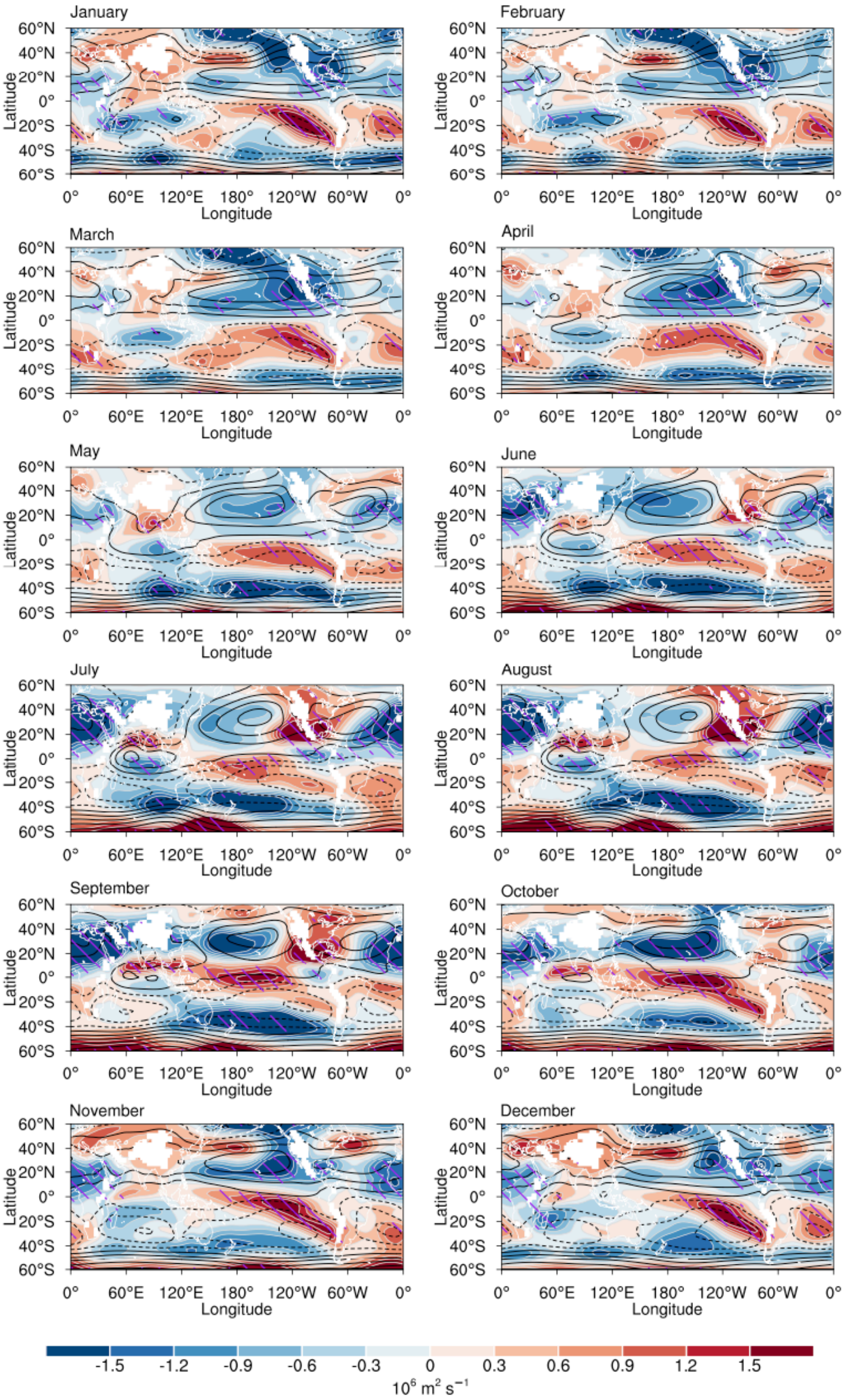

Figure S1. Changes in the 850 hPa streamfunction (shading) in the SSP5-8.5 experiment relative to the historical experiment in each month. The contours represent the climatology of the 850 hPa streamfunction in the historical experiment (interval: 5×10^{6} m^2 s^{-1}). Hatching indicates that the change is robust (see Methods for the details of the criteria).

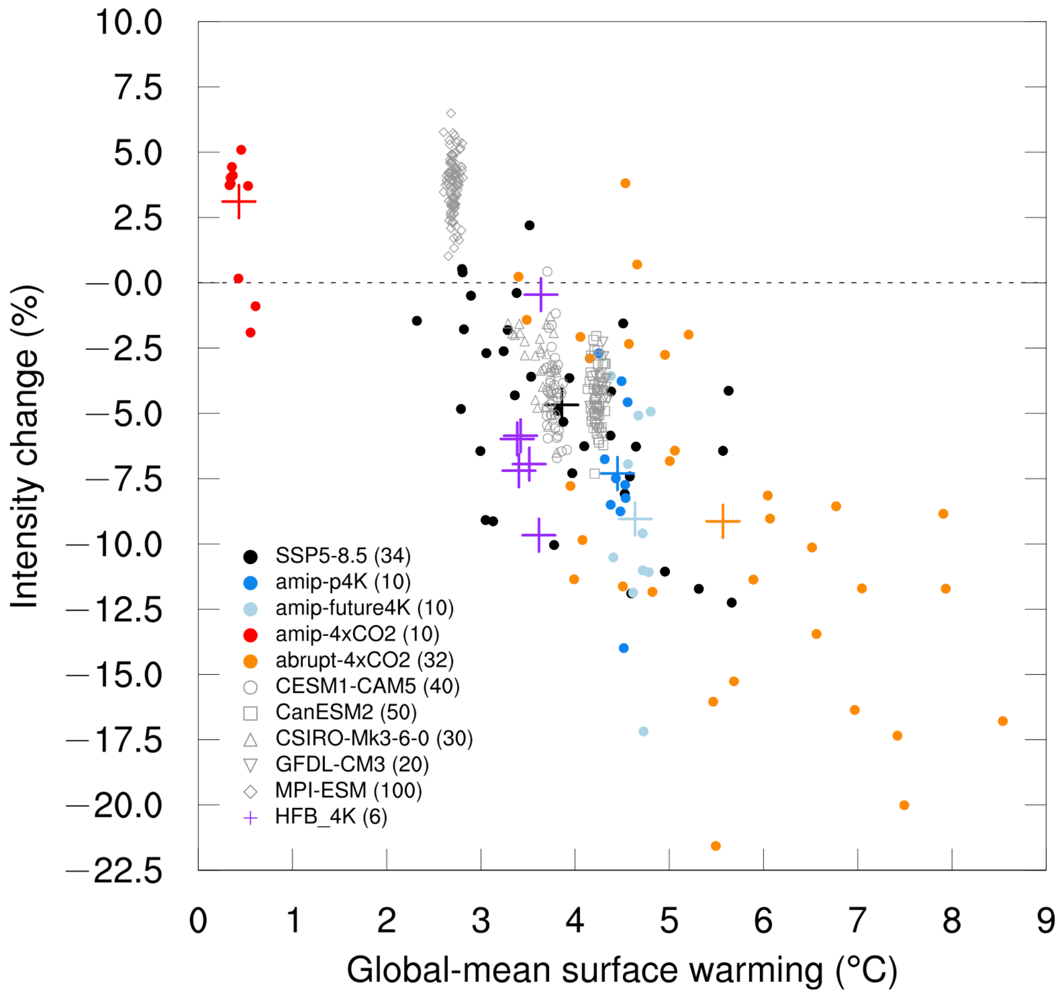

Figure S2. As in Fig. 2, but only for the Northern Hemisphere.

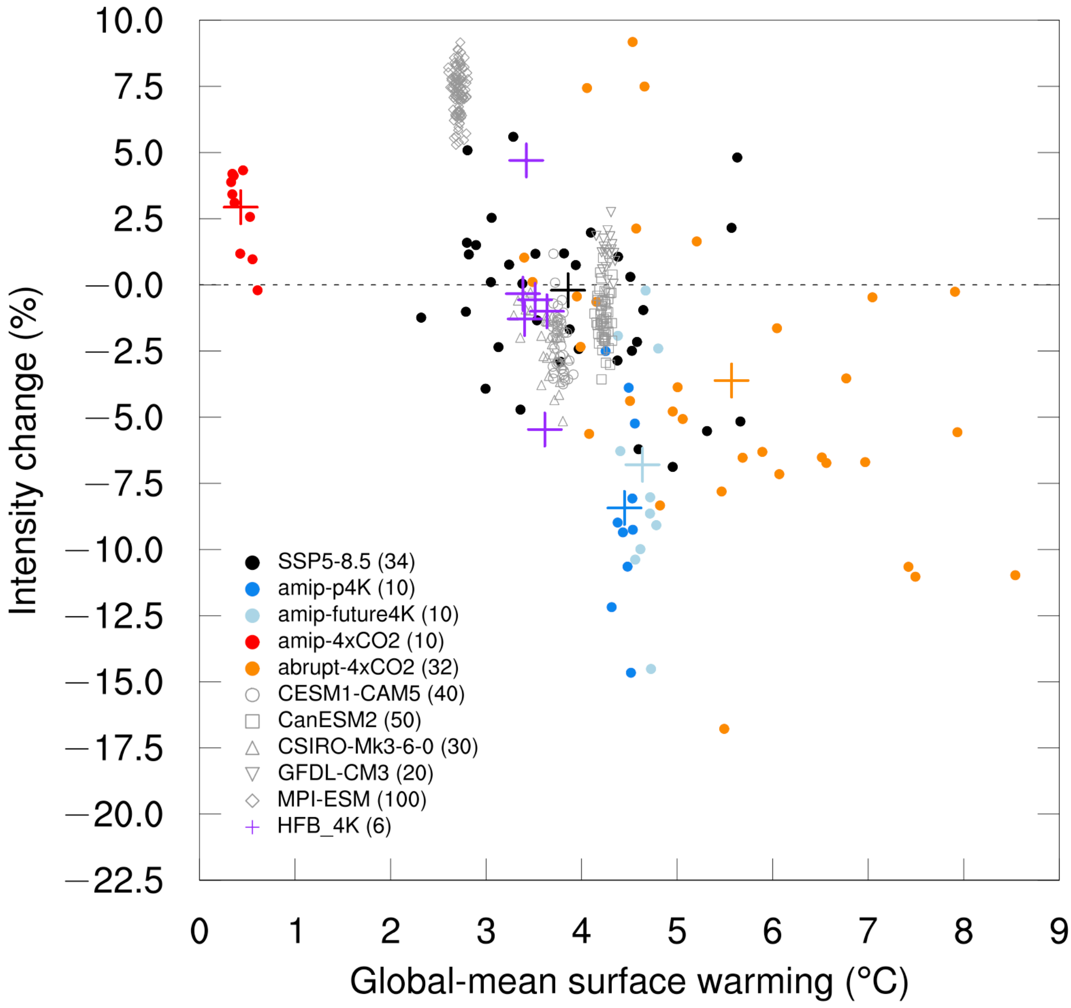

Figure S3. As in Fig. 2, but only for the Southern Hemisphere.

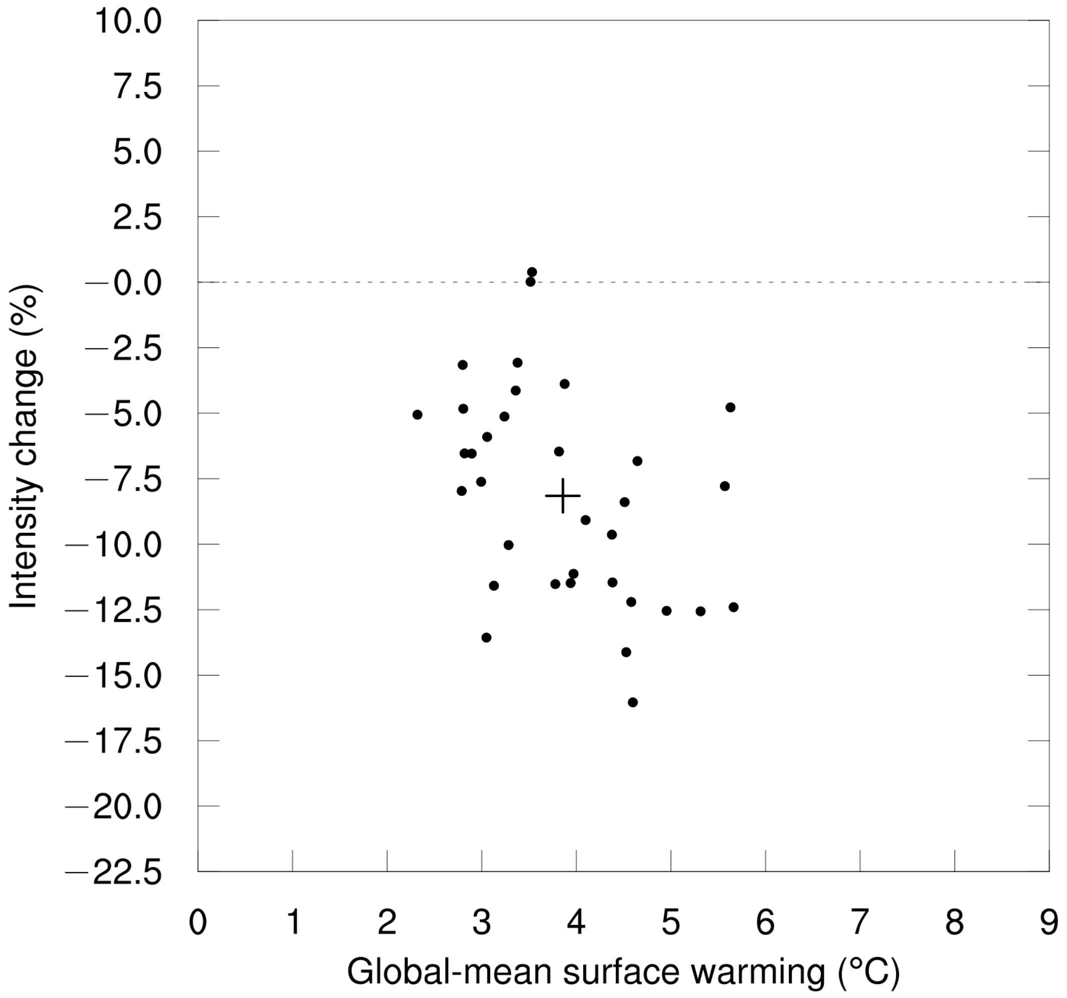

Figure S4. As in Fig. 2, but for the divergent component of subtropical circulation represented by the velocity potential at 850 hPa in the SSP5-8.5 runs.

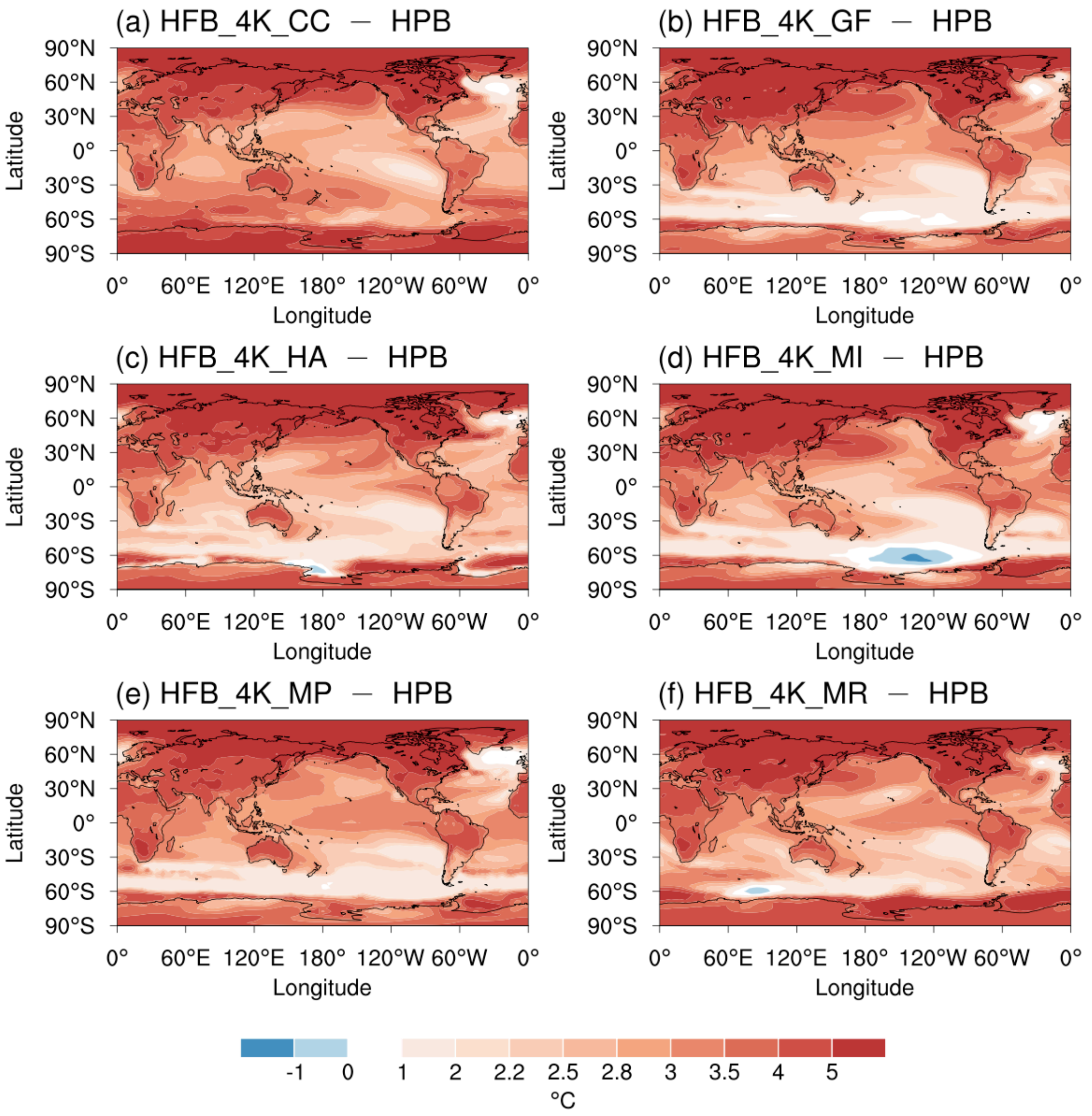

Figure S5. Annual-mean changes in the surface air temperature in the **a**, HFB_4K_CC (CCSM4), **b**, HFB_4K_GF (GFDL-CM3), **c**, HFB_4K_HA (HadGEM2-AO), **d**, HFB_4K_MI (MIROC5), **e**, HFB_4K_MP (MPI-ESM-MR) and **f**, HFB_4K_MR (MRI-CGCM3) experiments relative to the HPB (present climate) experiment from the d4PDF. There are 15 members for each future experiment. Only the results of the 15-member ensemble mean are shown here. Because the SST warming pattern is not provided in the d4PDF, the change in surface air temperature is shown here as a reference for the SST forcing pattern.

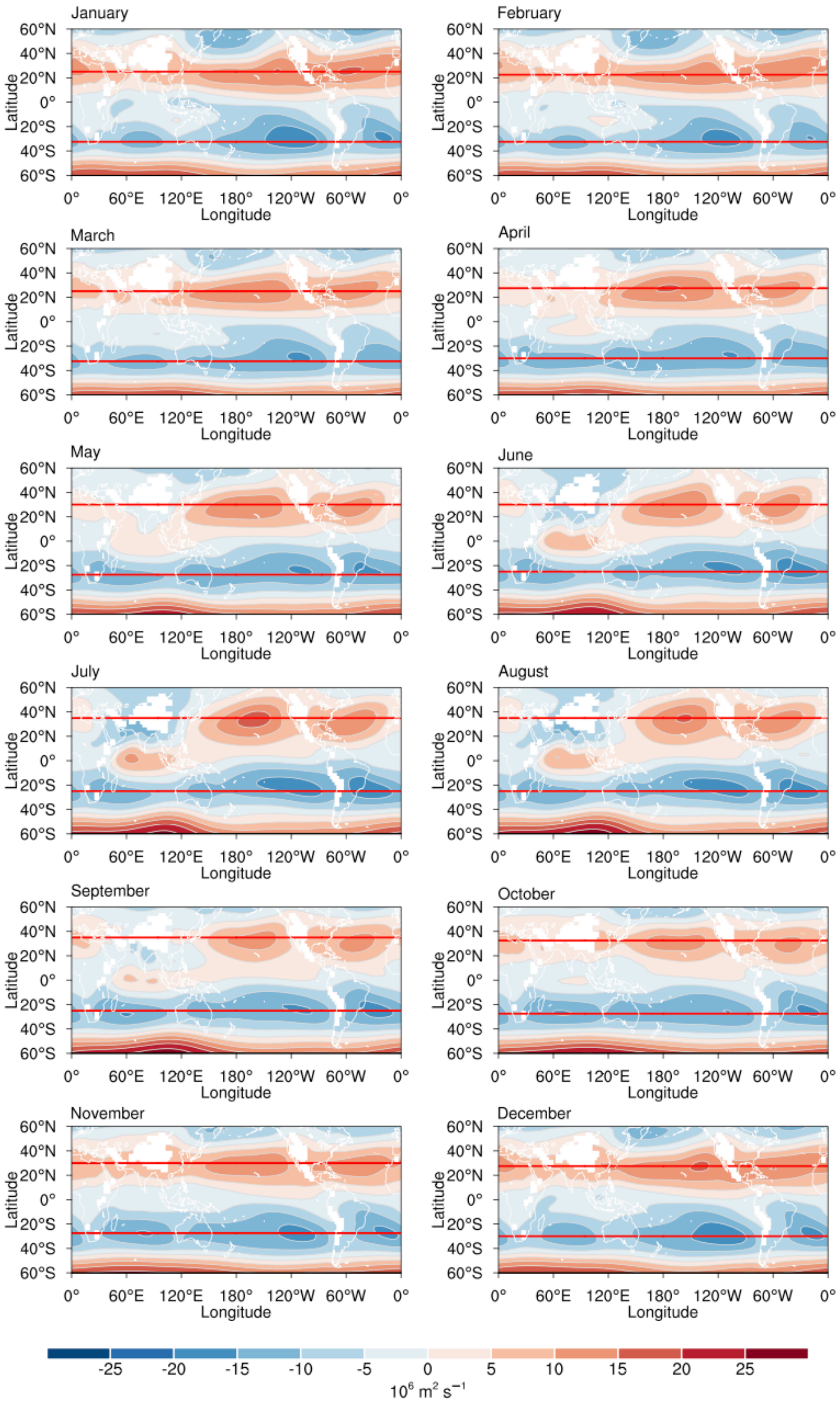

Figure S6. Monthly climatology of the 850 hPa streamfunction in the historical experiment. The red lines indicate the center of the subtropical circulation in each hemisphere (see Methods for details).

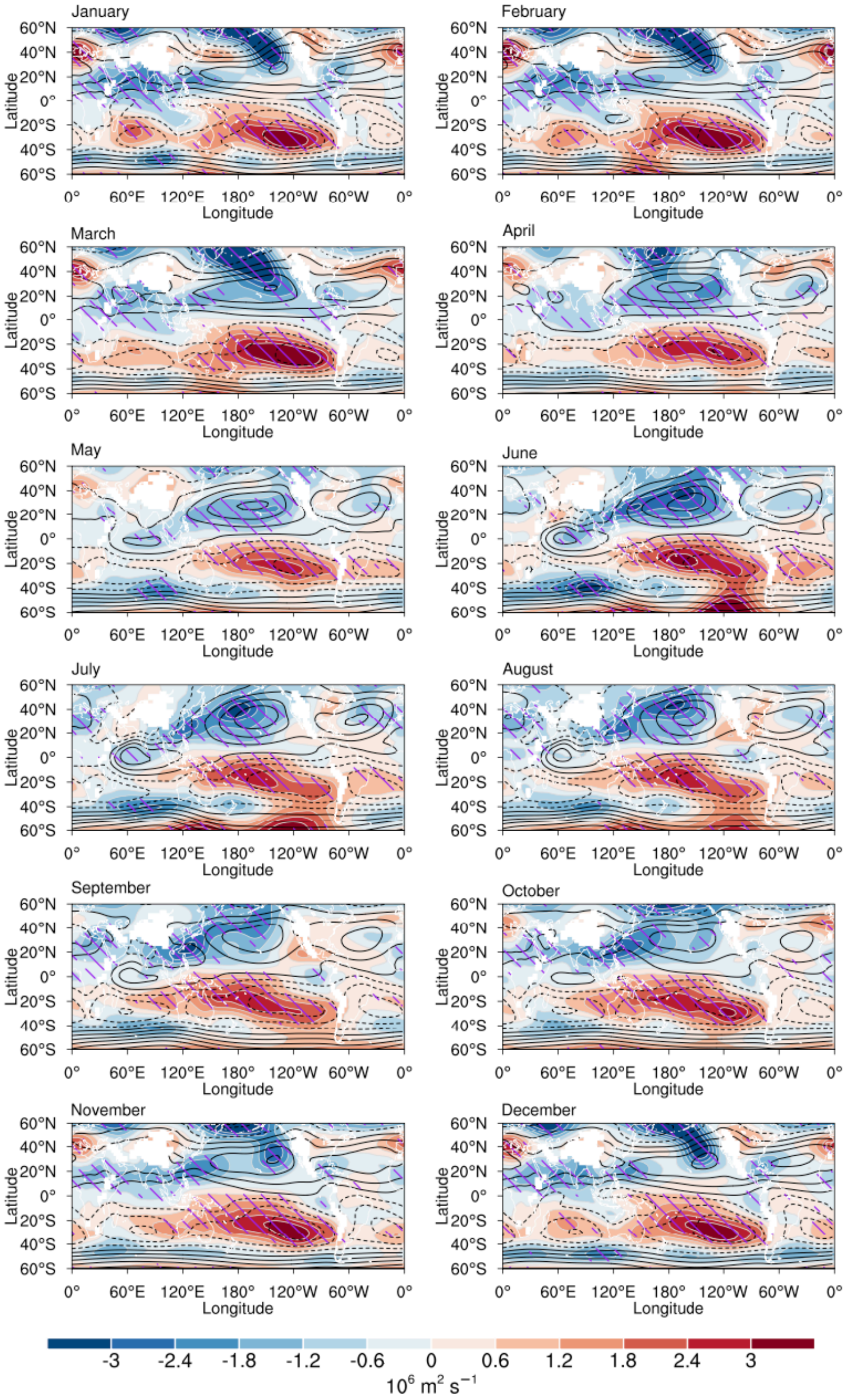

Figure S7. Changes in the 850 hPa streamfunction (shading) in the amip-p4K experiment relative to the amip experiment in each month. The contours represent the climatology of the 850 hPa streamfunction in the amip experiment (interval: 5×10^6 m^2 s^{-1}). Hatching indicates that the change is robust (see Methods for the details of the criteria).

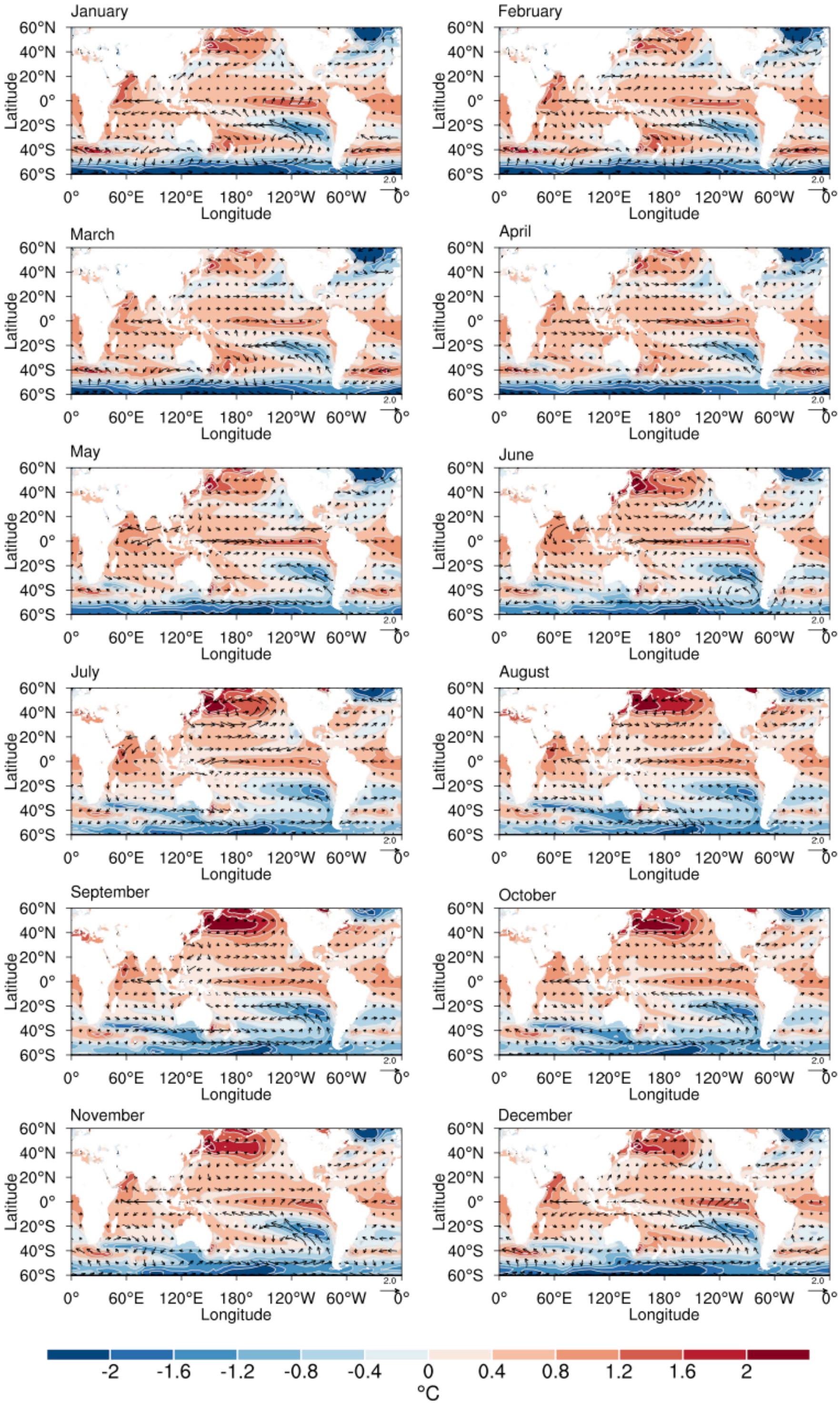

Figure S8. Changes in the sea surface temperature (shaded) and 850 hPa vector wind (vectors) in the amip-future4K experiment relative to the amip-p4K experiment in each month.

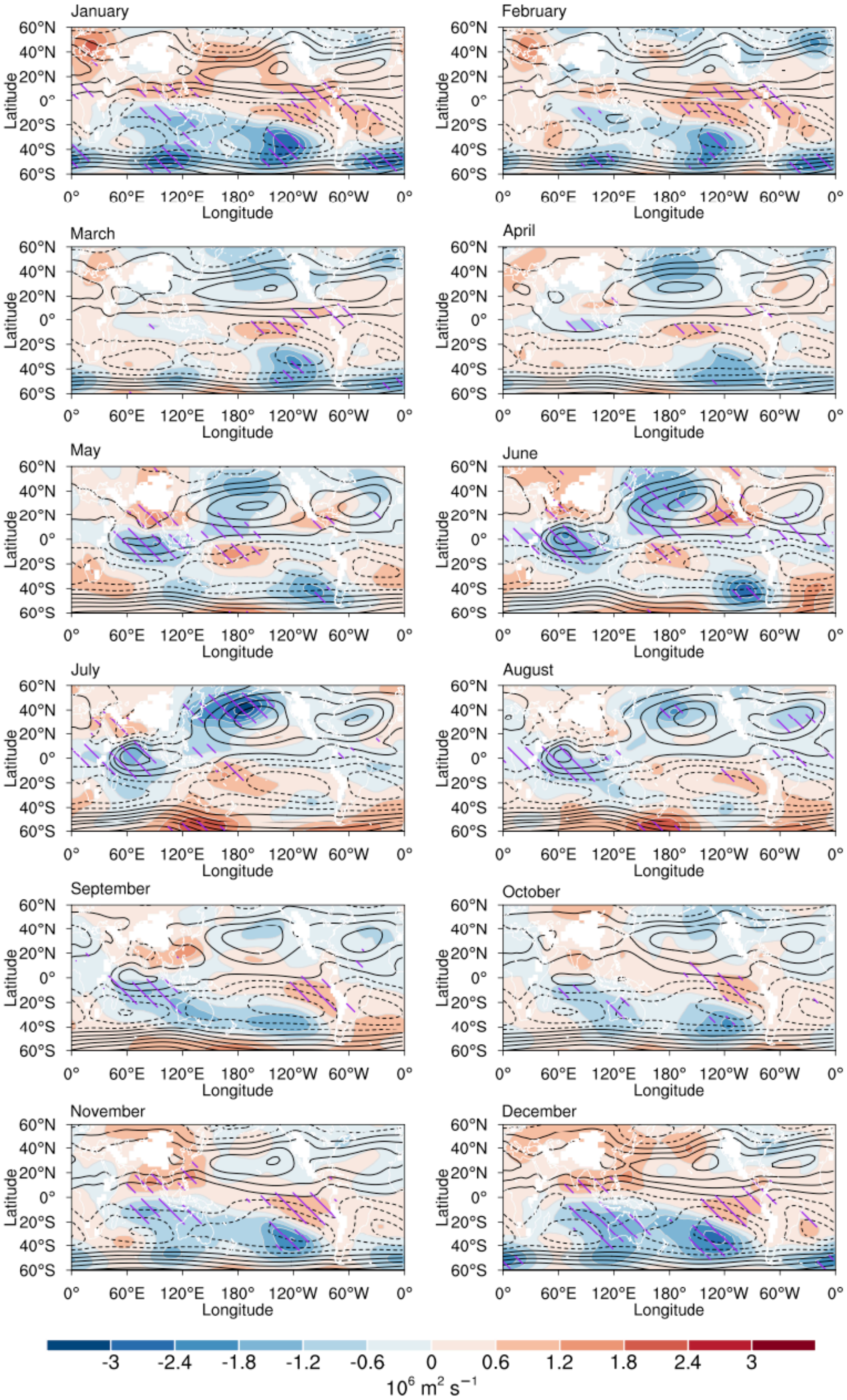

Figure S9. As in Fig. S7, but for the amip-future4K experiment relative to the amip-p4K experiment.

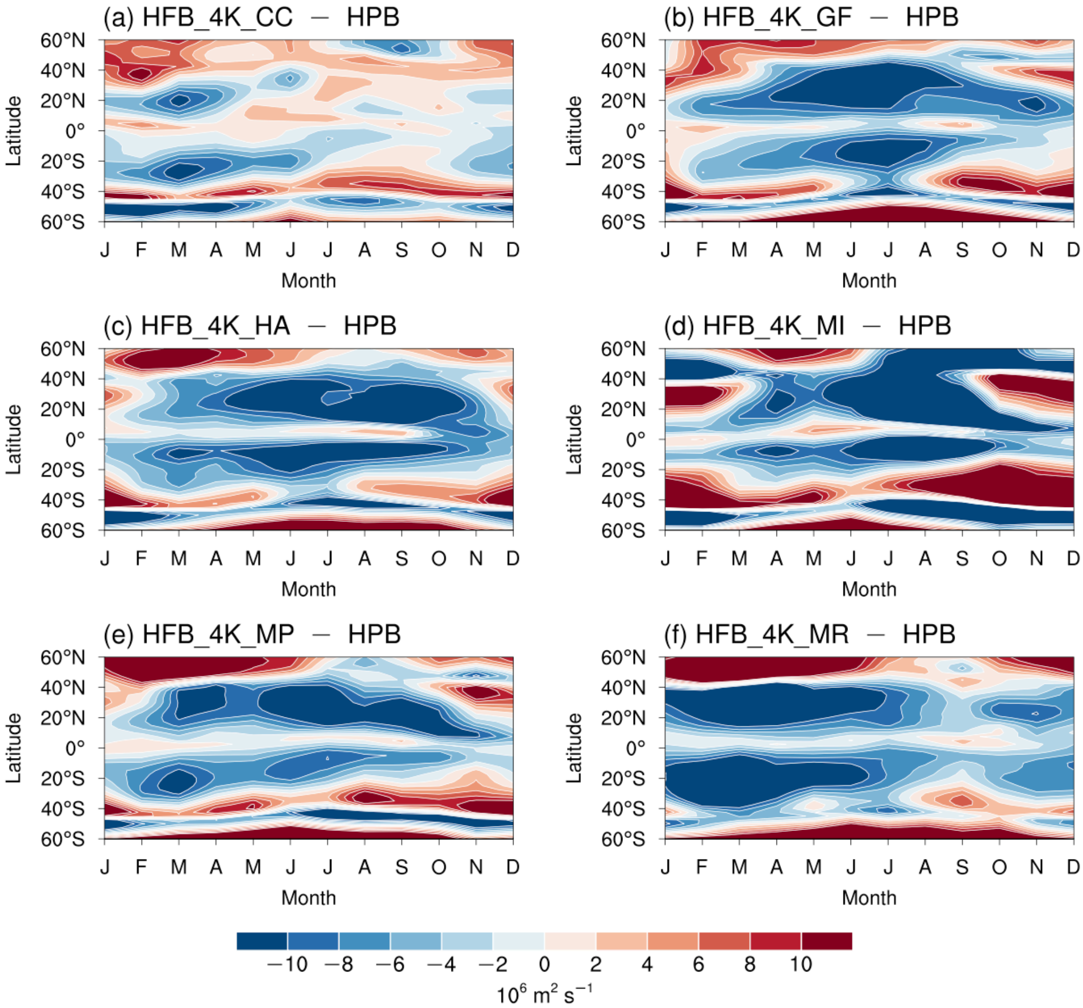

Figure S10. As in Fig. S5, but for the monthly and latitudinal intensity changes in 850 hPa streamfunction. There are 15 members for each future experiment. Only the results of the 15-member ensemble mean are shown at here.

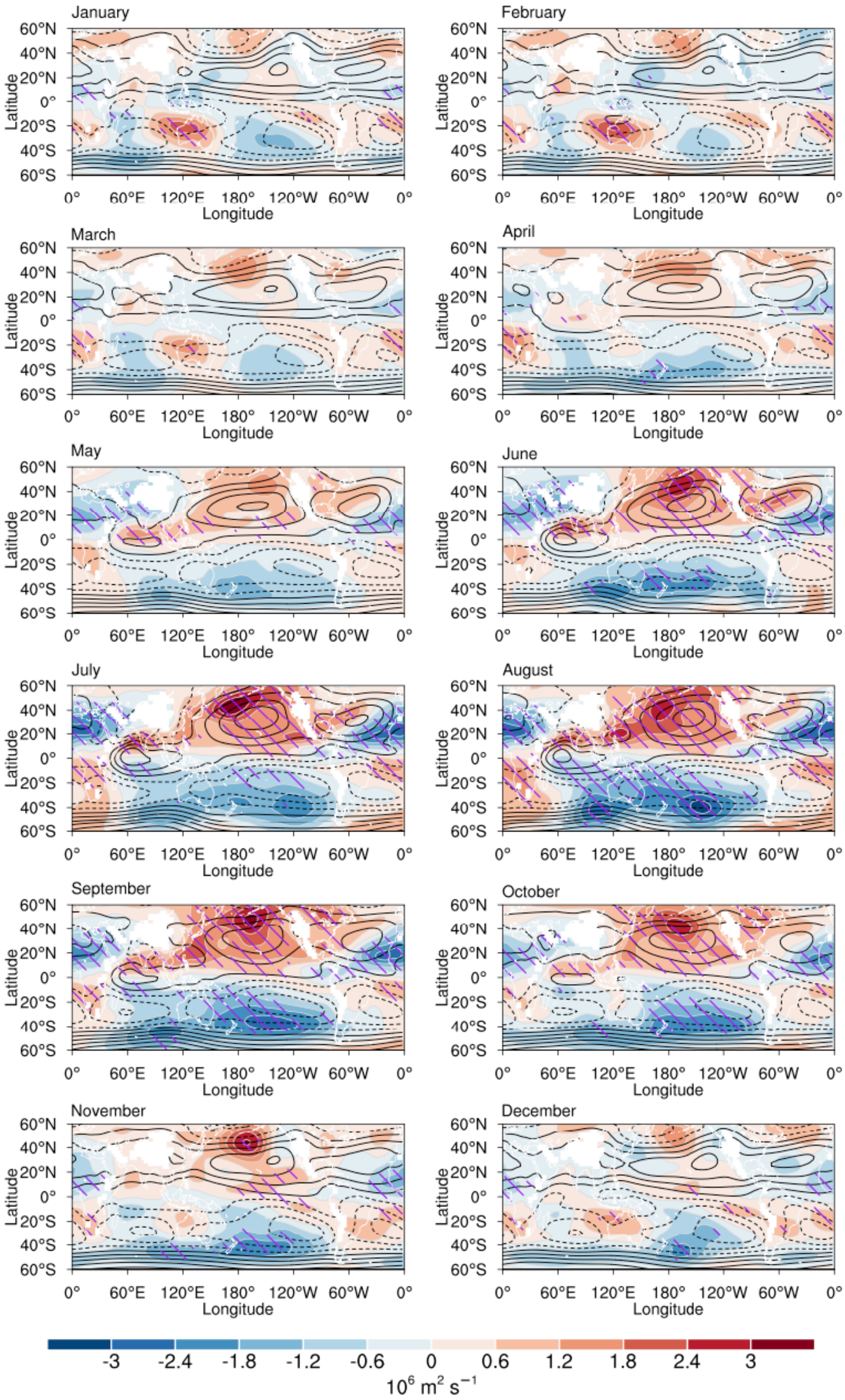

Figure S11. As in Fig. S7, but for the amip-4xCO2 experiment.

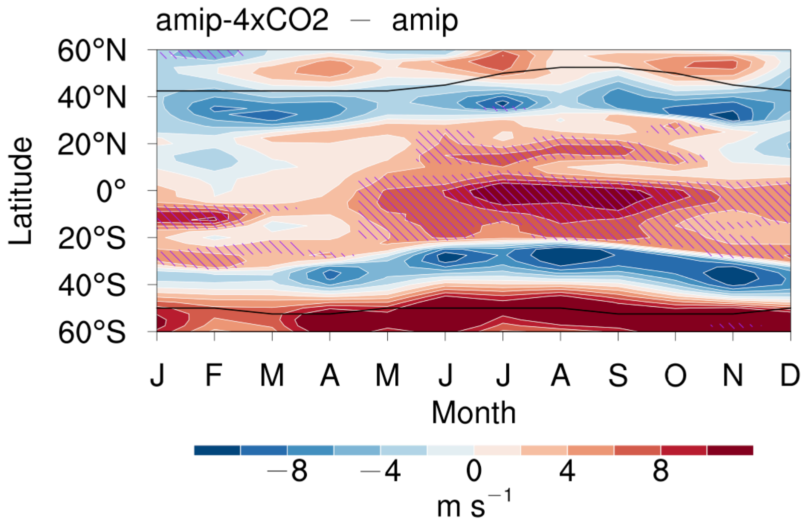

Figure S12. Seasonal changes in 850 hPa zonal wind (120°E–120°W) projected onto the climatology under direct CO_2 radiative forcing. The black curves indicate the maximum of zonal-mean 850 hPa climatological zonal wind in the amip experiment in each hemisphere. Hatching indicates that the change is robust (see Methods for the details of the criteria).

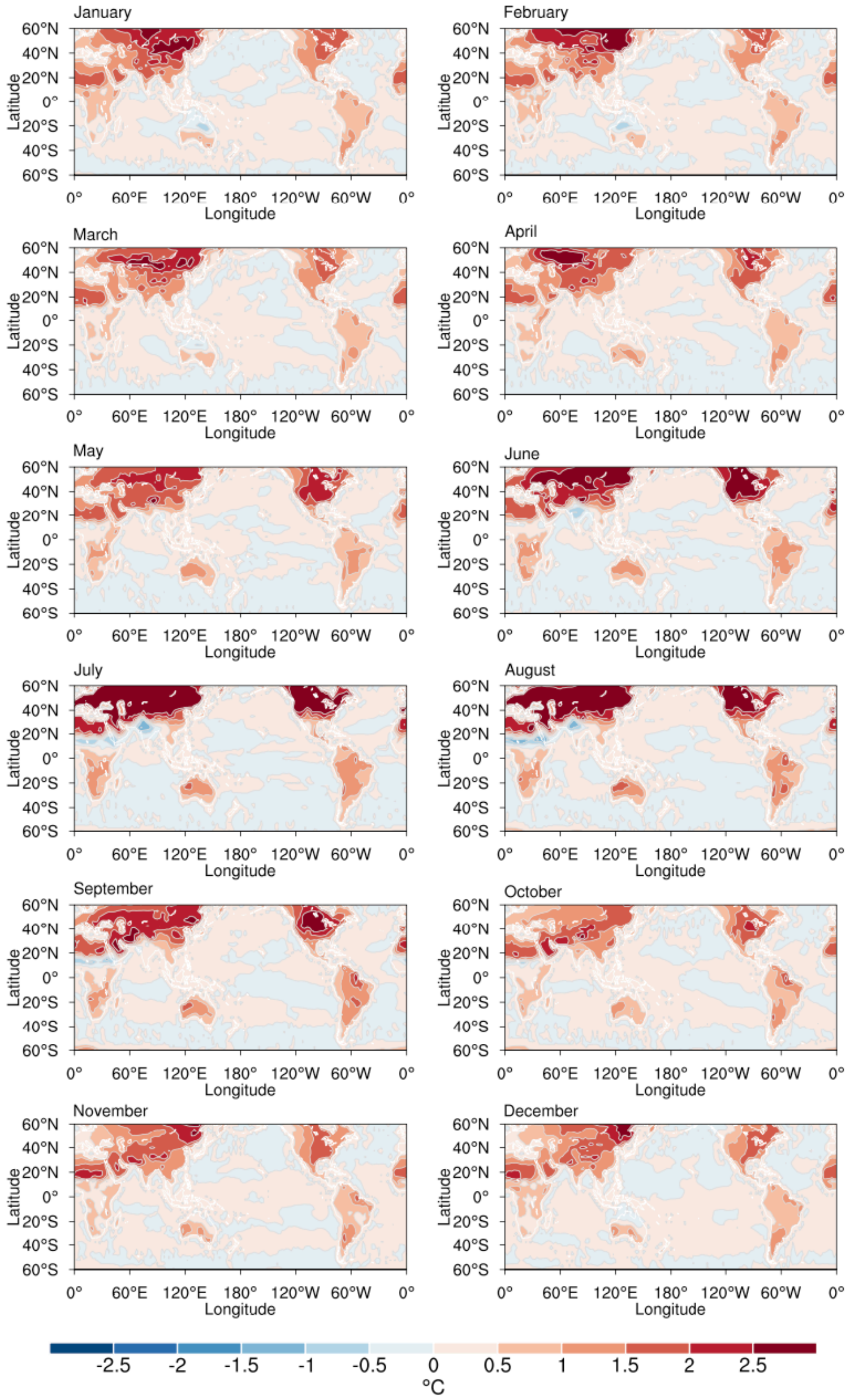

Figure S13. Changes in the surface temperature in the amip-4xCO2 experiment relative to the amip experiment in each month.

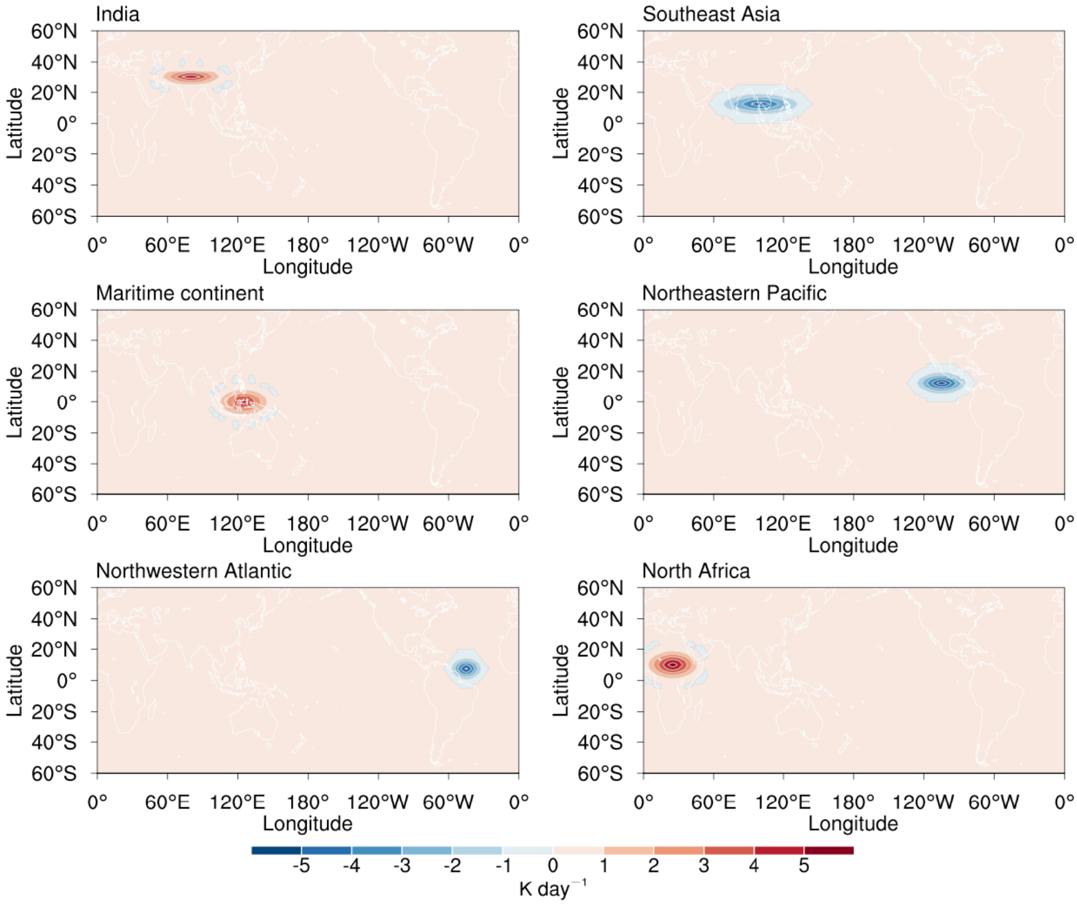

Figure S14. The prescribed diabatic heating and cooling for six regions used in the linear baroclinic model.

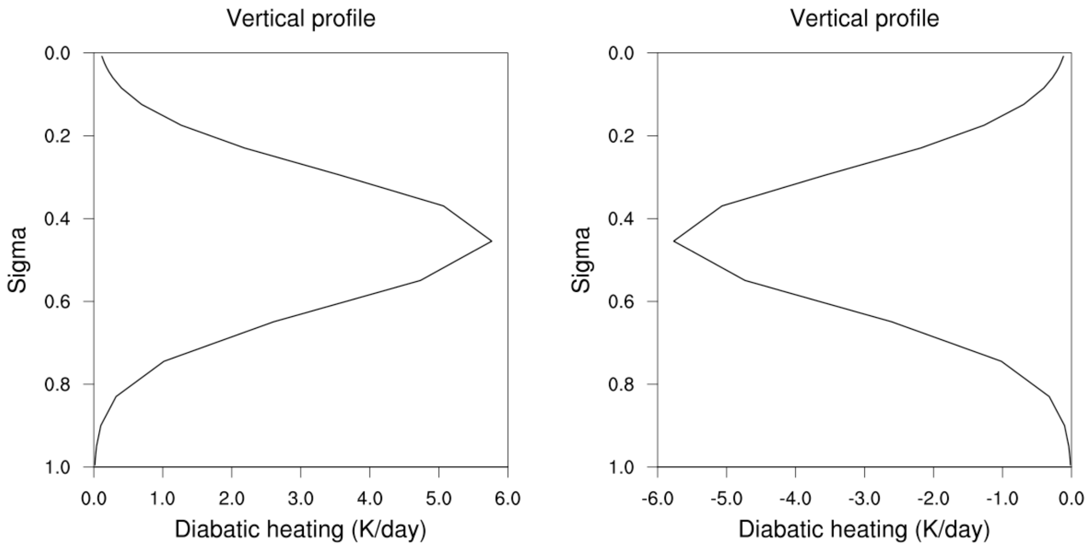

Figure S15. Vertical profile of the prescribed diabatic heating and cooling shown in Fig. S14.

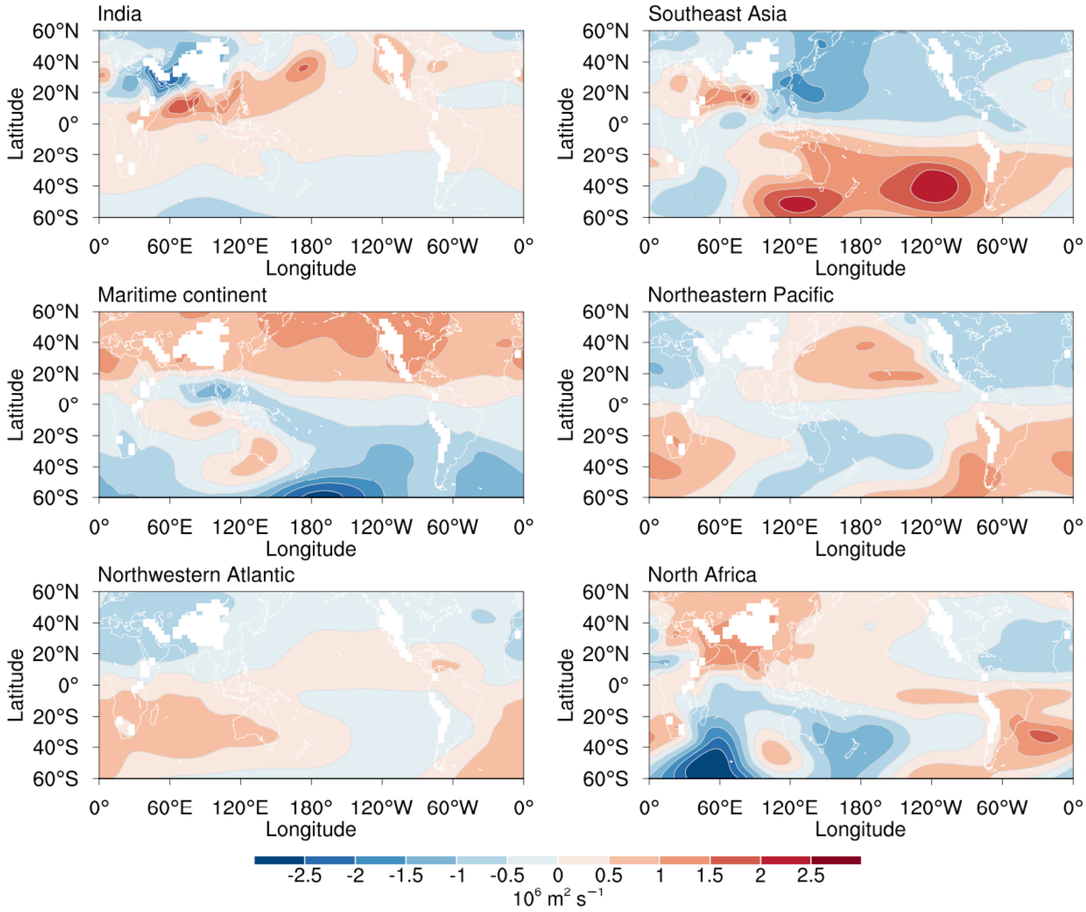

Figure S16. The steady response in 850 hPa streamfunction to the prescribed diabatic heating and cooling shown in Fig. S14.

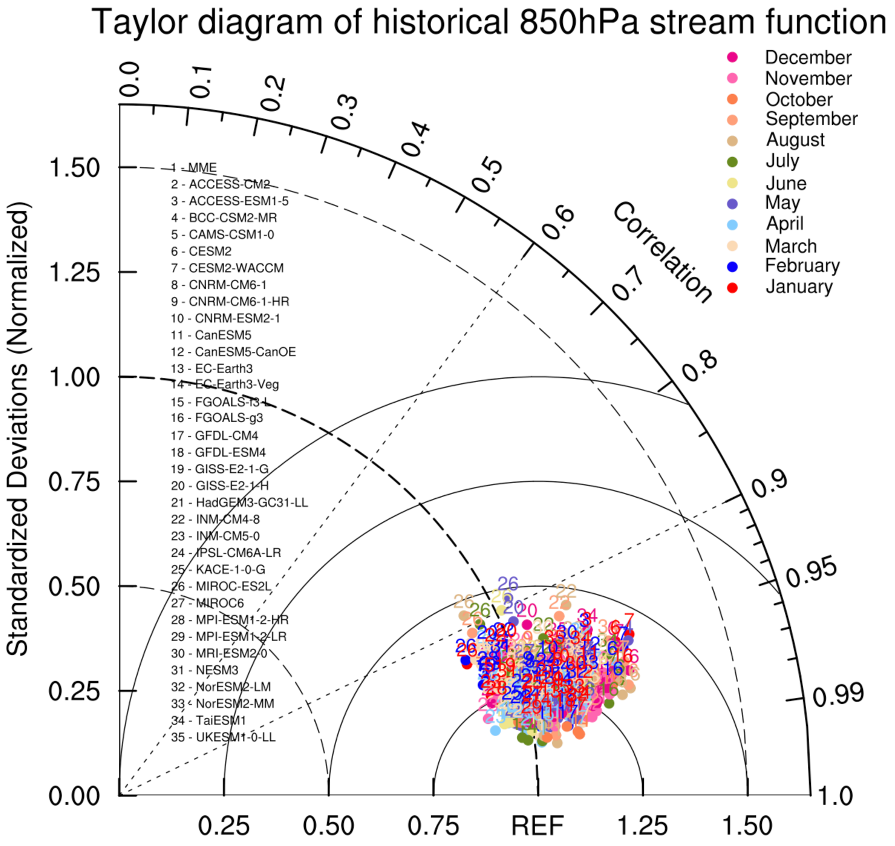

Figure S17. Taylor diagram of monthly climatology of the 850 hPa streamfunction in the historical experiment for 34 CMIP6 model covering 60°S–60°N with respect to data from ECMWF Reanalysis v5. The time span is from 1979 to 2008.

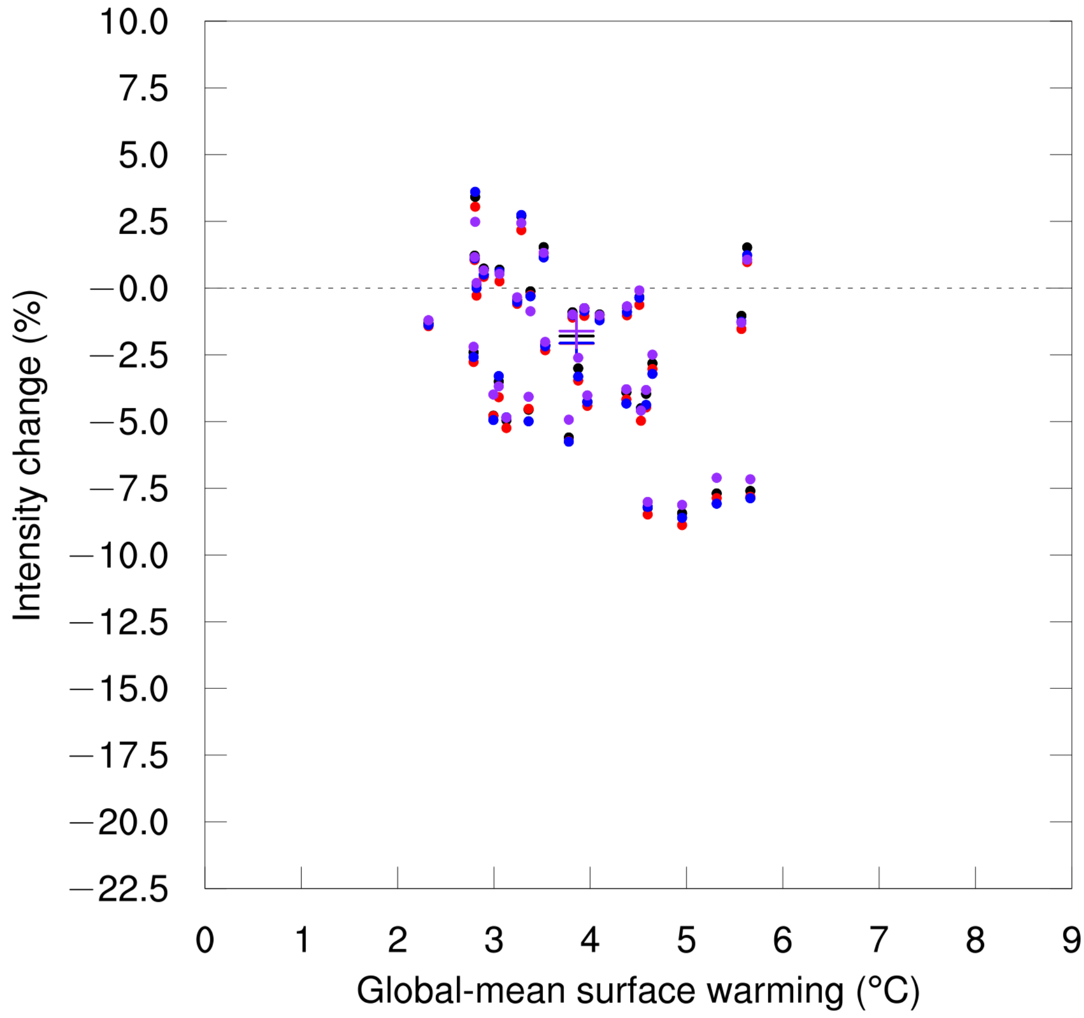

Figure S18. Intensity changes in the global subtropical circulation with respect to global-mean surface warming in SSP5-8.5. The intensity changes in the global subtropical circulation in SSP5-8.5 runs (years 2070–2099; 34 models) relative to historical runs (years 1979–2008) are calculated over 10°–45°S and 10°–45°N (black dots), 45°S–45°N (red dots), 10°–40°S and 10°–40°N (blue dots) and 10°–50°S and 10°–50°N (purple dots).
